# New Biomaterials and Regenerative Medicine Strategies in Periodontology, Oral Surgery, Esthetic and Implant Dentistry 2016

**DOI:** 10.1155/2017/8209507

**Published:** 2017-01-22

**Authors:** David M. Dohan Ehrenfest, Adriano Piattelli, Gilberto Sammartino, Hom-Lay Wang

**Affiliations:** ^1^Department of Oral and Maxillofacial Surgery, The University of Michigan Health System, Ann Arbor, MI, USA; ^2^Department of Periodontics and Oral Medicine, The University of Michigan, School of Dentistry, Ann Arbor, MI, USA; ^3^LoB5 Research Unit, School of Dentistry & Research Center for Biomineralization Disorders, Chonnam National University, Gwangju, Republic of Korea; ^4^Department of Medical, Oral and Biotechnological Sciences, University G. D'Annunzio of Chieti-Pescara, Chieti, Italy; ^5^Department of Oral Surgery, Faculty of Medicine, University Federico II of Naples, Naples, Italy

Biomaterial sciences are major fields of research in the current era of tissue engineering and regenerative medicine strategies, and these new developments define the new Frontier in many medical disciplines [1]. Biomaterials play a significant role in the expansion of regenerative treatments of the maxilla, to allow more sophisticated oral rehabilitation of edentulous patients [2, 3]. Periodontology, oral surgery, esthetic and implant Dentistry (the POSEID disciplines) are significant beneficiaries of these recent promising developments and these interconnected clinical domains are themselves major sources of research in implantable materials, particularly dental implants (new implant design, surfaces) [4], bone materials [2], or surgical adjuvants [3].

In the previous special issue focused on new biomaterials and regenerative medicine strategies in periodontology, oral surgery, esthetic and implant dentistry [1], we highlighted how these fields of research are deeply interconnected and represent major topics of transversal (multidisciplinary) and translational (from basic sciences to clinical applications) research, in order to develop new concepts and therapeutic strategies. In this 2016 edition of this issue, it is important to recall some ethical and legal issues related to this field.

In general, when referring to ethical issues, authors mostly limit their considerations to animal experiments, human trials, and their associated regulatory issues and need for Ethical Committee approval. However, regulatory issues are also very significant nowadays and should be considered with a lot of care as they have significant scientific consequences.

In the last years, the growth of the market of implantable materials in oral and maxillofacial applications (mostly the POSEID disciplines) has led to a dramatic and uncontrolled increase of the industrial producers and related stakeholders [5]. If we limit the discussion to dental implants and bone materials, the market evolved from a few major manufacturers to more than 500 manufacturers worldwide. On these official producers, one must count the numerous (at least the double) “pirate” producers, particularly active in some countries such as Brazil, Italy, or South Korea, that is, small manufacturers or retailers of small productions often operating without (or with limited) legal licenses (no CE marking, no FDA approval, etc.). Actually, many manufacturers, not only the pirates, are not legally up to date with their products, and serious controls by national health agencies would lead to a massive withdrawal of products (even in some major manufacturers in some cases) [6].

This situation appears as a strange paradox: drugs are very thoroughly investigated and controlled in most countries, but the control of biomedical devices and implantable materials appears very much neglected, particularly on oral and maxillofacial applications [6].

In front of this chaotic market, scientific and academic stakeholders have to be particularly careful with whom they collaborate and what they accept to publish in the international database. Many scientific articles available on Pubmed are in fact now investigating products that have no legal existence or remain at the borderline. Presented and appearing like “normally available products” in some scientific articles, some devices and materials that should not be on the market use the credibility of the journals to gain existence in the eyes of the readers.

Two interesting examples can be cited in 2016.

The first example is the permanent disorder on the dental implant market [5]: when investigating the evolution of the surfaces of the dental implants of a manufacturer year after year [7, 8], we can easily observe many significant variations of the production between years, sometimes even between batches produced in the same period [9]! The same observation can be raised when considering bone substitutes [2]. Many manufacturers are not following strict quality rules to standardize their production, which can lead to very poor quality products with batches contaminated with severe pollutions [9]; but many other manufacturers are in fact changing voluntarily the characteristics of their products during time, based on the feedback of clinicians, without informing the regulatory authorities of these changes; such changes revealing the weakness of the preclinical research normally needed to validate a product before being sold on the market [10]. For example, in the recent years, by tracking some products year by year, we could detect almost 12 significantly different versions of the implant surface from a major manufacturer, while these changes have never been disclosed or investigated.

The second example, particularly important in 2016, is related to the production of platelet concentrates for surgical use from the PRF (Platelet-Rich Fibrin) family [11]. Among those products, the most frequent material is classified as L-PRF (Leukocyte- and Platelet-Rich Fibrin) and is widely used in oral and maxillofacial applications [3, 12]. To use this method, there is only one system available on the market officially as a CE-marked and FDA-cleared device, based on the method that was developed 15 years ago and widely spread (Intra-Spin System, Intra-Lock, Boca-Raton, FL, USA) [11]. For a few years, many custom-made devices and protocols, using inadequate centrifuges, tubes, and devices, were used and sometimes marketed in many countries and different forms to produce this blood derivative, creating confusion and chaos in the mind of users. Some other variations of the original materials and techniques appeared also recently in the literature (e.g., the A-PRF system, so-called “Advanced Platelet-Rich Fibrin” as a trademark, low-cost and poor quality devices and tubes that are sold without any CE marking, FDA approval, or any forms of regulatory clearance) [13, 14], creating even more confusions for the readership. In fact, each change of the materials and protocols generates a new version of the PRF with different characteristics from the original L-PRF biomaterial, leading to nonreproducible results, mixed clinical outcomes in comparison to the original method, and finally major bias in the literature.

Facing this new threat for the credibility of the scientific literature and for their reputation, dental and biomaterial journals should make their standards of selection evolve and apply drastically the ethical standards used in high impact factor journals in the medical field, particularly to clarify systematically if the material of a research in an article is duly authorized, regulatory cleared, and standardized. Investigations on products, which have no legal existence in major markets, must be marked and clearly stated as experimental. In most major journals, to avoid this kind of disorder and confusions, authors have to fill a disclosure during the submission which is enforcing the regulatory issues related to tested products. For example, such texts are in use: “If your manuscript discusses an unlabeled use of a commercial product or device or an investigational use of a product or device not yet approved by the FDA for any purpose, you must specifically disclose in the manuscript that the product is not labeled for the use under discussion or that the product is still investigational.”

When observing the chaos growing with L-PRF technologies (and it is just starting) and their dramatic scientific and sanitary consequences, editors should force authors for such disclosure. It would be very beneficial for everyone.

These regulatory issues are not small administrative issues. The fact that tested products have no legal existence is a method of hijacking the scientific database for commercial purposes and is extremely damaging for the journal's credibility. But more important, the absence of regulatory controls also allows the development of unclear, undefined materials, with an absence of standardization of devices leading finally to unclear and biased scientific results. If the characteristics of biomaterials are not standardized, not declared in a legally binding way, and changing frequently without disclosure, what is the value of scientific results obtained with such materials [8, 11]?

As a conclusion, this 2016 special issue of new biomaterials and regenerative medicine strategies in periodontology, oral surgery, esthetic and implant dentistry continues its task to gather a meaningful corpus of relevant articles. But more than before, a better control of biomaterials placed on the market and of what is published in the specialized literature is needed.
